# Culmorin inhibits detoxification of the mycotoxin deoxynivalenol by plant UDP-glucosyltransferases

**DOI:** 10.1093/jxb/erag158

**Published:** 2026-03-28

**Authors:** Herbert Michlmayr, Gerlinde Wiesenberger, Katrin Rehak, Marta Sopel, Kristina Funtak, Alexandra Malachová, Philipp Fruhmann, Julia Weber, Marie Dufresne, Rudolf Krska, Franz Berthiller, Gerhard Adam

**Affiliations:** Department of Agricultural Sciences, BOKU University, Institute of Microbial Genetics, Tulln 3430, Austria; Department of Agricultural Sciences, BOKU University, Institute of Crop Breeding and Genomics, Tulln 3430, Austria; Department of Agricultural Sciences, BOKU University, Institute of Microbial Genetics, Tulln 3430, Austria; Department of Agricultural Sciences, BOKU University, Institute of Bioanalytics and Agro-Metabolomics, Tulln 3430, Austria; Department of Agricultural Sciences, BOKU University, Institute of Microbial Genetics, Tulln 3430, Austria; Department of Agricultural Sciences, BOKU University, Institute of Bioanalytics and Agro-Metabolomics, Tulln 3430, Austria; Department of Agricultural Sciences, BOKU University, Institute of Microbial Genetics, Tulln 3430, Austria; Department of Agricultural Sciences, BOKU University, Institute of Bioanalytics and Agro-Metabolomics, Tulln 3430, Austria; FFoQSI GmbH Austrian Competence Centre for Feed and Food Quality, Safety and Innovation, Tulln 3430, Austria; Institute of Applied Synthetic Chemistry, TU Wien, Vienna 1060, Austria; Institute of Applied Synthetic Chemistry, TU Wien, Vienna 1060, Austria; Université Paris-Saclay, INRAE, UR BIOGER, Palaiseau 91120, France; Department of Agricultural Sciences, BOKU University, Institute of Bioanalytics and Agro-Metabolomics, Tulln 3430, Austria; Institute for Global Food Security, School of Biological Sciences, Queen’s Untiversity Belfast, Belfast BT9 5DL, Northern Ireland, UK; Department of Agricultural Sciences, BOKU University, Institute of Bioanalytics and Agro-Metabolomics, Tulln 3430, Austria; Department of Agricultural Sciences, BOKU University, Institute of Microbial Genetics, Tulln 3430, Austria; INRAE-Bordeaux, France

**Keywords:** Culmorin, deoxynivalenol, *Fusarium*, glucosylation, inhibition kinetics, mycotoxin, plant–pathogen interaction, root, secondary metabolism, sesquiterpene, trichothecene, virulence, wheat

## Abstract

The *Fusarium* metabolite culmorin (CUL) frequently co-occurs with the mycotoxin deoxynivalenol (DON) on cereals. While DON is recognized as a major *Fusarium* virulence factor on plants, the function of CUL is still unclear. In this study, we show that CUL-deficient *F. graminearum* mutants created by deletion of the terpene synthase gene *CLM1* are less aggressive on wheat than the wild-type, and this is accompanied by increased DON-3-glucoside/DON ratios in infected wheat ears. In root elongation assays with wheat and *Brachypodium distachyon*, CUL had no effect alone but significantly increased the toxicity of DON. Analysis of roots treated with DON and/or CUL further indicated that both wheat and *B. distachyon* are able to glucosylate CUL and that its presence impedes DON-glucosylation in both species. We identified two *B. distachyon* UDP-glucosyltransferases (UGTs) able to glucosylate CUL, and further investigated the effects of CUL on the kinetics of validated DON-glucosylating plant UGTs (BdUGT5g03300, HvUGT13248, OsUGT79). This suggested that CUL inhibits DON-glucosylation either by serving as a competitive substrate with DON or by unproductive binding. BdUGT5g03300 in particular was strongly inhibited by CUL and even its glucosides. Our results indicate that CUL contributes to *Fusarium* virulence by weakening plant defences related to UGT-catalysed DON-detoxification. As even CUL-glucosides are potentially inhibitory to UGTs, this implies a complex synergy of CUL with DON.

## Teaser

The *Fusarium* metabolite culmorin contributes to virulence on plants by suppressing detoxification of the virulence factor deoxynivalenol by glucosyltransferases.

## Introduction

Infection by *Fusarium* species is a global threat to cereal crops such as wheat, barley, and maize, causing drastic yield losses and deterioration of quality due to mycotoxin contamination (Goswami and Kistler, 2004; Chen *et al*., 2019). The (sesquiterpene) trichothecene mycotoxin deoxynivalenol (DON) inhibits eukaryotic protein synthesis and is a crucial virulence factor of *F. graminearum*, required for spreading on wheat ears (Proctor *et al*., 1995; Desjardins *et al*., 1996; Bai *et al*., 2002). DON is a highly problematic food contaminant and due to its ubiquitous occurrence it is one of the economically most important mycotoxins. Acute or chronic exposure cause several clinical symptoms in humans and animals (Pestka, 2007; Pestka, 2010). Legislation or guidance values have therefore been put in place in many regions of the world, for instance in the United States of America (US Food & Drug Administration, 2010), the European Union (The European Commission, 2023), and several Asian countries (Anukul *et al*., 2013).

The tricyclic sesquiterpene diol culmorin (CUL) is produced by several *Fusarium* species including *F. graminearum*, *F. crookwellense*, *F. venenatum*, and the name-giving *F. culmorum* (Pedersen and Miller, 1999). Upon its first description (Ashley *et al*., 1937), it received little attention due to its low apparent toxigenic properties; however, several later studies indicated that CUL persistently co-occurs with DON in equivalent or even higher concentration levels (Ghebremeskel and Langseth, 2001; Streit *et al*., 2013; Uhlig *et al*., 2013; Hajnal *et al*., 2020; Radić *et al*., 2021), raising concerns regarding its function and toxicological potential. Weak anti-fungal activity of CUL against several marine and terrestrial (human pathogenic) fungi has been reported (Strongman *et al*., 1987). *In vitro* assays have further implied low toxicity to animal cells (Gruber-Dorninger *et al*., 2017), with no negative effects being observed on the growth of piglets (Rotter *et al*., 1992) and *Heliothis zea* larvae (Dowd *et al*., 1989). However, CUL significantly increases mortality of the latter when applied in combination with DON, and Dowd *et al*. (1989) were the first to propose a synergistic effect with DON due to competition for detoxification enzymes.

As sesquiterpenes, CUL and trichothecene class toxins such as DON share farnesyl diphosphate as a biosynthetic precursor. The trichodiene synthase encoded by *TRI5* catalyses the first step in trichothecene biosynthesis by converting farnesyl diphosphate to trichodiene (McCormick *et al*., 2011). Disruption of *TRI5* in *F. graminearum* abolishes DON synthesis and results in reduced virulence (Proctor *et al*., 1995). Synthesis of culmorin occurs in two steps, starting with the conversion of farnesyl diphosphate to longiborneol by the terpene synthase CLM1 (McCormick *et al*., 2010). The cytochrome P450 CLM2 catalyses the subsequent hydroxylation of longiborneol to CUL (Bahadoor *et al*., 2016). Both *CLM1* and *CLM2* are induced under trichothecene-inducing conditions (McCormick *et al*., 2010; Sieber *et al*., 2014; Harris *et al*., 2016; Wipfler *et al*., 2019). Although this suggests a role in virulence, evidence remains scarce. A phytotoxic effect of CUL has been reported on wheat coleoptile tissue at high concentrations (1000 µM CUL; Wang and Miller, 1988). However, at concentrations closer to natural contaminations levels (10 µM, equivalent to ∼3 mg l^−1^) CUL has no phytotoxicity on wheat by itself but synergistically increases the toxicity of DON and several other trichothecene toxins (Wang and Miller, 1988). Wipfler *et al*. (2019) also reported synergistic effects of CUL and DON on wheat, barley, and maize. The severity of Fusarium head blight (FHB) disease on wheat caused by 15 *F. graminearum* isolates is positively correlated with the sum of CUL and DON but negatively correlated with the CUL/DON ratio (Wipfler *et al*., 2019).

The aim of the present study was to further investigate the effect of CUL on wheat and *Brachypodium distachyon*, in particular by addressing its potential interaction/synergy with DON. *Brachypodium distachyon* is a monocot grass with a small genome and short life cycle, and it is amenable to genetic transformation (Scholthof *et al*., 2018). It is a host species to many cereal pathogens (Pasquet *et al*., 2016) and a useful model system to study FHB. Following *F. graminearum* infection, it exhibits typical FHB symptoms including accumulation of DON in infected spikes (Peraldi *et al*., 2011; Pasquet *et al*., 2014).

We created CUL-deficient *F. graminearum* strains to test whether the ability to synthesize CUL has an impact on virulence on wheat. We further investigated the combined effect of CUL and DON on wheat and *B. distachyon* roots. The results of these experiments implied that CUL impairs detoxification of DON to DON-3-glucoside in plants, and hence we also conducted steady state kinetic assays to investigate whether CUL can biochemically interfere with the detoxification of DON by previously validated DON-conjugating plant UDP-glucosyltransferases.

## Materials and methods

### Deoxynivalenol and culmorin

DON was purified as described by Altpeter and Posselt (1994). CUL and its conjugated derivatives were synthesized and purified as described by Weber *et al*. (2018).

### Plant material

The FHB-sensitive wheat (*Triticum aestivum* 2*n*=6*x*=42, AABBDD) cultivar Apogee, a dwarf variety with a short flowering cycle (Bugbee *et al*., 1997; Mackintosh *et al*., 2006), was used for infection experiments and root assays. The *Brachypodium distachyon* lines used were Bd21-3 (wild type) and OE-10R14, and the TILLING line 6829-7. OE-10R14 is a Bd21-3 line over-expressing the UDP-glucosyltransferase (UGT) BdUGT5g03300 under the *Zea mays* ubiquitin promoter (Pasquet *et al*., 2016). The TILLING line 6829-7, carrying a W345* mutation in the *BdUGT5g03300* gene (in the region encoding the PSPG box) was previously identified by screening the *B. distachyon* TILLING mutant collection BRACHYTIL (Dalmais *et al*., 2013; Pasquet *et al*., 2016). The TILLING line 6829-3 that carries the wild-type *Bradi5g03300* allele was selected as a control line (Pasquet *et al*., 2016).

### Fungal strains, cultivation, and construction of knockout strains

*Fusarium graminearum* PH-1 (NRRL 31084, FGSC 9075) was grown routinely on Fusarium Minimal Medium (Leslie and Summerell, 2006) and sporulated in mung bean broth (10 g mung beans per litre of water). Conidiospores were quantified by counting them in a Fuchs–Rosenthal chamber. For determination of mycotoxins, strains were cultivated in 2-SM medium [3 g l^−1^ KH_2_PO_4_, 0.2 g l^−1^ MgSO_4_.7H_2_O, 5 g l^−1^ NaCl, 1 g l^−1^ (NH_4_)_2_HPO_4_, 40 g l^−1^ sucrose, 10 g l^−1^ glycerol, 10 mg l^−1^ citric acid, 10 mg l^−1^ ZnSO_4_.6H_2_O, 2 mg l^−1^ Fe(NH_4_)_2_(SO_4_)_2_.6H_2_O, 0.5 mg l^−1^ CuSO_4_.5H_2_O, 0.1 mg l^−1^ MnSO_4_, 0.1 mg l^−1^ H_3_BO_4_, 0.1 mg l^−1^ Na_2_MoO_4_.2H_2_O). Magenta™ vessels containing 20 ml of 2-SM medium were inoculated with 10^5^ spores and incubated at 20 °C in the dark for 3 weeks. Mycelia were separated from the medium using a Buchner funnel fitted with a filter paper, and flow-through was analysed by LC-MS/MS (see below).

For deletion of the *CLM1* (FGSG_10397) gene in PH-1 two constructs were produced. First, the 3′-flanking region of the gene was amplified using the primer pair AATGTCGACTATGACAAACCAGATCTGAGTAAC and TACAAGCTTGAAGACGATCAGCTGCATCCTA, the resulting amplicon was digested with SalI and HindIII, and the 532 bp fragment was then ligated to pKT245 (Twaruschek *et al*., 2018) cleaved with the same enzymes. The resulting plasmid was termed pKF33. Similarly, the 5′-flanking region was amplified using the primers TTATCTATTAGGCCGCATAGGCCTGATTG and TAAACTAGTGTCTATATTCCGGCTGTAGATCA and the fragment was cloned into pKT245 via SfiI and SpeI, yielding pKF65. Next, pKF33 was digested with HindIII, RsrII, and SacII, and pKF65 with SfiI and PstI to release fragments of 3128 bp and 2074 bp, respectively. Fragments containing either the 3′- or the 5′-flanking region plus non-functional parts of the *nptII* selection marker were mixed in a 1:1 molar ratio and 10 µg of this DNA mix were used to transform PH-1 as described by Twaruschek *et al*. (2018), and transformants were selected on 40 mg l^−1^ Geneticin (G418, Sigma-Aldrich). Five transformants with the correct DNA integration (*clm1*Δ::*loxP*-HSVtk-*nptII*-*loxP*) were identified by PCR analysis, and two of these were used for further work (KFCUL-37 and KFCUL-41; Supplementary Fig. S1). By treating these strains with CRE-recombinase the HSVtk-*nptII* marker cassette was removed, yielding strains GWCUL-37.37 and GWCUL-41.41 (*clm1*Δ::*loxP*). A control, ‘PH-1t’, was regenerated from diluted protoplasts of a mock transformation with no DNA and no selective agent.

### Wheat infection

Seeds of wheat cv. Apogee were sterilized with 1.4% (w/v) NaOCl, and 0.06% (w/v) Triton-X-100 for 10 min with shaking and subsequently washed twice with sterile H_2_O. For germination, the seeds were placed on wet (9 ml of half-strength Murashige-Skoog medium without sucrose) filter papers in 145×20 mm Petri dishes (∼15 seeds per dish) and incubated for 24 h in a growth chamber set to 20 °C, 75% relative humidity, and a cycle of 18/6 h light/dark. Plants were grown in an autoclaved mixture of soil and perlite (3:1) until flowering (20 °C, 22/2 h light/dark, 75% relative humidity; Watson *et al*., 2018) and were watered at 2–3 d intervals, with one addition of fertilizer at 3–4 weeks after sowing. Infection with *F. graminearum* was carried out by inoculating four flowers per ear with 10 µl each of a spore suspension containing 4×10^4^ conidia ml^−1^. Ten ears were treated per strain (PH-1, PH-1t, GWCUL-37.37 and GWCUL-41.41). The infection experiment was performed independently three times and, starting at day 4 post-infection, progression of disease symptoms was assessed every second day until day 14, 10, and 12, respectively. On the last day, the ears were cut off the stems, frozen in liquid nitrogen, and stored at −80 °C until subsequent use. The ears were ground using a Retsch Ball Mill MM 400 and aliquots of the powder were weighed into 1.5 ml tubes. A 10-fold volume of 50% MeOH was added and the samples were extracted on a Vibrax shaker (IKA, Staufen, Germany) for 30 min at room temperature. After centrifugation for 15 min at 21 130 *g* and 4 °C the samples were appropriately diluted with methanol/water (1:1) and analysed by LC-MS/MS (see below).

### Infection of *B. distachyon* line OE-10R14

The OE-10R14 line of *B. distachyon* was cultivated in a growth chamber with a 20/4 h light/dark cycle at 23 °C ±2 °C under fluorescent light (265 µE m^−2^ s^−1^ at soil level and ∼315 µE m^−2^ s^−1^ at the spike level). Prior to sowing, seeds were surface-sterilized by incubation in a 0.6% sodium hypochlorite solution for 10 min with gentle shaking followed by three rinses in sterile distilled water. The seeds were subsequently incubated for 5 d at 4 °C in the dark. Plants were grown on a 2:1 mixture of compost (Tref terreau P1; Jiffy France SARL, Trevoux, France) and standard perlite and soaked with an aqueous solution containing a carbamate fungicide (Previcur at 2 ml L^−1^; Bayer Crop Sciences) and a larvicide (Hortigard at 1 g L^−1^; Syngenta France). Plants were routinely watered at 2–3 d intervals using a standard nutritional solution (NPK: 14-12-32, Plant-Prod, Ferti SAS, Boulogne-Billancourt, France) and were never allowed to stand in water.

Inoculation was performed by spraying whole plants with fungal spore suspensions (10^5^ conidia ml^–1^) until dripping. The inoculated plants were covered with clear plastic bags, the interiors of which had been sprayed with distilled water beforehand. For the first 24 h, the inoculated plants were kept in the dark, after which they were kept under a photoperiod of 16/8 h light/dark at 20 °C at the same light intensity as for plant development described above. Symptoms were evaluated at 7 d and 14 d post inoculation. A spikelet was considered symptomatic if at least half of its florets were symptomatic.

For quantification of *F. graminearum* DNA, two biological replicates each consisting of four spray-inoculated spikes pooled per strain sampled 2 weeks after infection were used. Quantification of fungal DNA was performed by qPCR on 10 ng of total DNA using primers specific for the genomic region encoding the 18S ribosomal subunitas previously described (Pasquet *et al*., 2016). For analysis of DON, DON-3-glucoside (D3G), CUL, and CUL-glucosides, 50 mg of pooled infected spikes (same samples as used for DNA quantification) were extracted with 500 µl of 50% methanol for 30 min at 25 °C and then analysed by LC-MS/MS (see below).

### Root elongation assays

Murashige–Skoog (MS) medium (Murashige and Skoog, 1962) was prepared without sucrose. Growth assays were performed in half-strength MS without sucrose with 0.5% (w/v) agarose (SERVA Electrophoresis GmbH, Heidelberg, Germany) containing DON and/or CUL at 5 mg l^–1^. CUL was dissolved in 1% (v/v) DMSO, and therefore all media including the controls were adjusted to the same DMSO concentration. The assays were done in 200 µl wide orifice pipette tips (Denville Scientific, Metuchen, NJ) filled with 360 µl of agarose medium. The tips were placed into flat bottom micro-inserts (0.2 ml, 31×6 mm) for HPLC vials (VWR, Darmstadt, Germany). Seeds of Apogee were prepared and germinated as described above and ‘planted’ into the agarose when the roots were clearly developed but less than 5 mm long, and then grown at 20 °C, 75% relative humidity with a light/dark cycle of 18/6 h. Images of the experimental set-up are shown in Supplementary Fig. S2. After 96 h, the roots were cut from the plants, washed to remove agarose residues, scanned at a resolution of 400 dpi using an Epson Perfection V700 Photo scanner, and the total root length was determined using the WinRHIZO Pro 2013 software (Regent Instruments Inc., Québec, Canada).

Seeds of *B. distachyon* seeds were sterilized with 0.7% sodium hypochlorite (NaOCl) for 20 min by gentle shaking (80 rpm) at room temperature, then washed three times with sterile H_2_O. The seeds were then stratified in the dark at 4 °C for 1–2 weeks. For germination, the seeds were placed on sterile damp filter paper in 145×20 mm Petri dishes (∼25 seeds per dish), kept in the dark at 4 °C for 3 d, and then the temperature was increased to 20 °C (still in the dark). Seedlings were transferred to agarose when the primary roots were ∼5 mm long. The agarose concentration was reduced to 0.3% to make it easier to penetrate the medium without injuring the roots. Most plants developed one-to-three adventitious roots, with the majority developing two. After 6 d, the total root length was determined as described above.

### Extracts from roots exposed to mycotoxin

Seedlings of Apogee and *B. distachyon* with roots 1–2 cm long were incubated in full-strength MS without sucrose and containing 50 mg l^−1^ DON and/or either 50 mg l^−1^ CUL (Apogee) or 10 mg l^−1^ CUL (*B. distachyon*). The seedlings were placed into sterile 2 ml tubes containing 500 µl medium such that only the roots were submerged. After 24 h incubation under the same conditions as specified above for the elongation assays, the roots were cut from the seedlings, washed with 10% ethanol, briefly dried with tissue paper, and weighed. The roots were then ground in the Retsch Ball Mill MM 400 for 1 min at 30× s^−1^ using two 5 mm steel balls, after which 500 µl methanol was added and the roots were extracted for 2 h at room temperature. The concentrations of DON, D3G, CUL, and CUL-glucosides were determined by LC-MS/MS.

### LC-MS/MS determination of DON, CUL, and their glucosides

DON, D3G, CUL, and CUL-glucosides were measured on a 6500+ QTrap MS/MS instrument (Sciex) coupled to an Agilent 1290 UHPLC system. A Kinetex C18 column (150×2.1 mm, 2.6 µm particle size; Phenomenex, Torrance, USA) was used for separation at 30 °C. Mobile phases consisted of mixtures of methanol and water (A, 10% MeOH; B, 97% MeOH) and both contained 5 mM ammonium acetate. A flow rate of 250 µl min^–1^ and an injection volume of 1 µl were chosen. The gradient started with 0% B for 1.0 min and then increased linearly to 100% B at 8.5 min. The column was rinsed with 100% B for 3 min, before equilibration with the start conditions for another 3 min until the end of the run at 14.5 min. The switching valve of the mass spectrometer directed the eluent into the ion source between 3.0 min and 11.0 min. The IonDrive Turbo V electrospray source (Sciex) was operated at 550 °C with 50 psi of gas 1 and gas 2, 30 psi of curtain gas, and ionization voltages of +4500 V in positive and −4500 V in negative mode. DON and D3G were detected in negative ion mode first, and after 4.5 min the polarity was changed to positive electrospray mode to detect CUL and CUL-glucosides. The dwell time for each selected reaction monitoring (SRM) transition was set to 50 ms with a pause time between transitions of 5 ms. The following SRM transitions were used for CUL: *m*/*z* 256.2 > 221.2 [collision energy (CE) 17 eV] and *m*/*z* 256.2 > 203.2 (CE 21 eV) using the [M+NH_4_]^+^ ion as the precursor with a declustering potential (DP) of 70 V. For CUL-glucosides the [M+NH_4_]^+^ ion was also used as the precursor (DP 70 V) with the following SRM settings: *m*/*z* 418.2 > 177.3 (CE 35 eV) and *m*/*z* 418.2 > 221.2 (CE 30 eV). For DON the [M+CH_3_COO]^−^ ion was used as the precursor (DP −70 V) with the following SRM transitions: *m*/*z* 355.1 > 265.1 (CE −22 eV) and *m*/*z* 355.1 > 59.1 (CE −40 eV). D3G was determined with the [M+CH_3_COO]^−^ ion as the precursor (DP −80 V) with the following SRM settings: *m*/*z* 517.2 > 427.1 (CE −30 eV) and *m*/*z* 517.2 > 59.1 (CE −85 eV). Retention times were 3.21 min for D3G, 3.37 min for DON, 8.31 min for CUL-glucosides, and 8.91 min for CUL. Using these chromatographic conditions, CUL-8-glucoside and CUL-11-glucoside could not be separated, and they are referred to collectively as CULGs.

### Glucosyltransferase assays

*Oryza sativa* OsUGT79 (NM_001058779), *B. distachyon* BdUGT5g03300 (KQJ81826.1), and *Hordeum vulgare* HvUGT13248 (ADC92550.1) were expressed with *E. coli* and purified as described by Michlmayr *et al*. (2018). Assays with DON and CUL were conducted at 25 µM substrate concentration at 25 °C and contained 10 mM UDP-glucose (uridine 5′-diphosphoglucose disodium salt hydrate from *Saccharomyces cerevisiae*; Sigma-Aldrich) and 100 mM potassium phosphate buffer, pH 7.0 (assay volume ∼100–200 µl). After 10–15 min, the assays were stopped by adding a 10-fold volumetric excess of methanol. The kinetics of HvUGT13248 with CUL were assayed under the same conditions using 5, 12.5, 25, 50, 125, and 250 µM CUL. The stopped assay samples were stored at 4 °C for subsequent quantification of DON, D3G, CUL, and CULGs by LC-MS/MS after 1:10 dilution in methanol. Reaction rates were calculated as µmol or nmol glucoside formed per min and mg of protein. All assays were conducted in triplicate.

### Steady-state kinetics and kinetic models

Steady-state enzyme kinetics were measured using a coupled enzymatic reaction to detect released UDP by NADH oxidation (Wetterhorn *et al*., 2017). In a total assay volume of 40 µl, the reaction mixture contained 100 mM Tris·HCl (pH 7.0), 2 mM phosphoenolpyruvate, 0.2 mM NADH (both Roche), 5 mM MgCl_2_, 2 µl pyruvate kinase/lactic acid dehydrogenase from rabbit muscle (Sigma-Aldrich), 1 mM UDP-glucose, and DON in different concentrations up to 16.9 mM. If required, UGTs were diluted in 100 mM Tris·HCl (pH 7.0) in order to obtain a linear time-dependent response. CUL was dissolved in methanol and added to the reaction mix from a 50-fold stock solution. Reactions were started by addition of DON dissolved in H_2_O, with blanks containing H_2_O instead of DON. Assays were carried out in 384-well plates, and the reactions were monitored by reading the absorbance at 340 nm in an EnSpire 2300 Multilabel Reader (PerkinElmer) adjusted to 25 °C. Enzyme reaction rates were calculated from the decrease in NADH using a calibration curve measured under similar conditions (pH, buffer, temperature) and expressed as µmol min^−1^ mg^−1^ referring to the release of UDP per mg of one-step purified protein.

The (apparent) parameters *V*_max_ and *K*_M_ were estimated by iterative curve fit with SigmaPlot 11.0 using the Michaelis–Menten model (Equation 1) or the Haldane model of substrate inhibition (Equation 2). Inhibition by culmorin was analysed according to a classic approach using Lineweaver–Burk diagrams following Bisswanger (2015). The corresponding models of competitive, uncompetitive, and non-competitive (mixed) inhibitions are shown in Equations 3, 4, and 5, respectively. The inhibitor constants *K*_ic_ (competitive) and *K*_iu_ (uncompetitive) were estimated from secondary diagrams plotting the slopes and *y*-axis intercepts of the primary Lineweaver–Burk regressions versus inhibitor concentration [I]. The *x*-intercept (*y*=0) of the regression in the secondary plot corresponds to the negative value of the respective *K*_i_: plotting the slopes versus [I] yields –*K*_ic_, and plotting *y*-intercepts versus [I] yields −*K*_iu_.

(1) Michaelis–Menten model:    $v=\;\frac{V_{\max}\;\times \;[S]}{K_{M}\;+[S]}$
(2) Substrate inhibition:    $v=\frac{V_{\max}\;\times \;[S]}{K_{M}\;+\;[S]\;+\;\frac{{\left[S\right]}^{2}}{K_{i}\;}}$
(3) Competitive inhibition:    $v=\;\frac{V_{\max}\;\times \;[S]}{K_{M}\;+\;\frac{[I]\;K_{M}}{K_{\mathrm{ic}}\;}\;+\;[S]}$
(4) Uncompetitive inhibition:    $v=\;\frac{V_{\max}\;\times \;[S]}{K_{M}\;+\;\left(1+\;\frac{\left[I\right]}{K_{\mathrm{iu}}\;}\right)\;\times \;[S]}$
(5) Full kinetic model of mixed inhibition: $v=\;\frac{V_{\max}\;\times \;[S]}{K_{M}\;\times \;\left(1+\;\frac{\left[I\right]}{K_{\mathrm{ic}}}\right)\;+\;\left(1+\;\frac{\left[I\right]}{K_{\mathrm{iu}}\;}\right)\;\times \;[S]}$

where [S] is the substrate concentration.

### Statistical analysis

All statistical tests were performed in R Studio. Infection scores and root-length assays were tested for normality with the Shapiro–Wilk test, and the homogeneity of variances was tested with the Levene test implemented in the car package for R (Fox and Weisberg, 2019). Since each dataset contained at least one group that deviated from normal distribution, all datasets were analysed using the Kruskal–Wallis rank sum test implemented in R. Post-hoc analyses for pairwise comparisons were conducted with the Wilcoxon rank sum test using the Benjamini and Hochberg (1995) method to adjust for multiple testing. Letter-based representations of pairwise comparisons were created with the function ‘multcompLetters’ implemented in the library ‘multcompView’ (Piepho, 2004). Relative area under disease progression curve (rAUDPC) was calculated with the R package ‘agricolae’. Paired *t*-tests (two-sample, two-tailed) were used to compare analytical results.

## Results

### Deletion of *CLM1* reduces the virulence of *F. graminearum* PH-1 on wheat

**Fig. 1.**
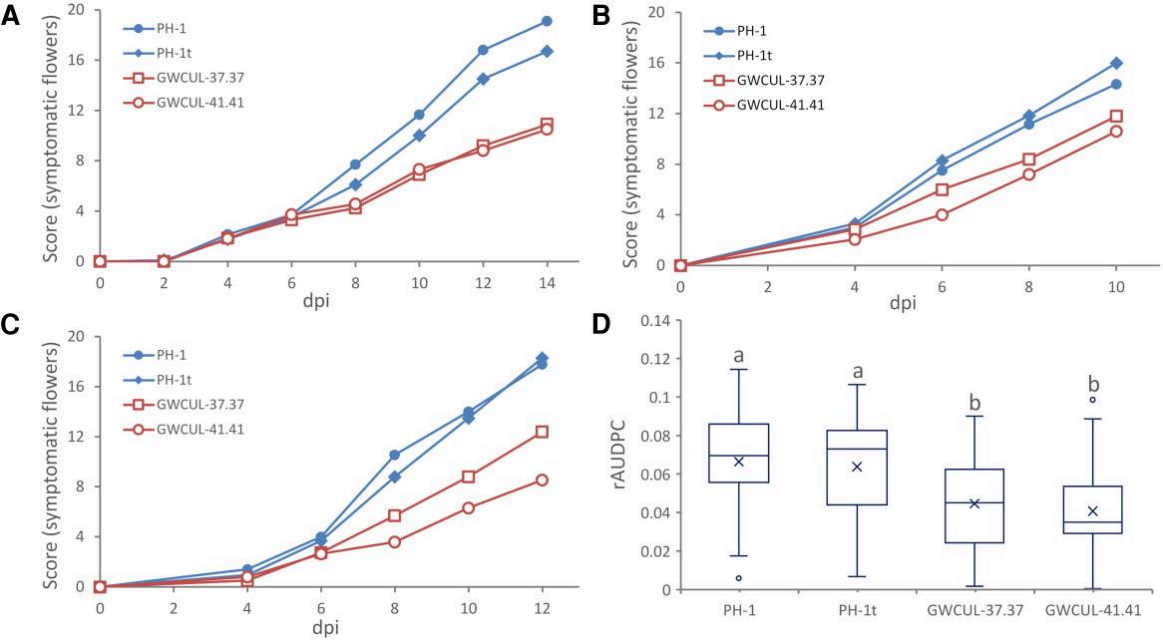
Disease progression on ears of wheat cultivar Apogee infected with the *Fusarium graminearum* wild type and *clm1*Δ mutant strains deficient in culmorin (CUL). GWCUL-37.37 and GWCUL-41.41 are the mutant strains and PH-1t is a mock-transformed control line. Four flowers per ear were infected. (A–C) Variation with time in the number of symptomatic flowers per ear for three independent experiments; dpi, days post infection. Data are means of *n*=10 ears. (D) Comparison of time-standardized ‘relative area under the progression curve’ (rAUDPC) for all experiments pooled.; × denotes the arithmetic mean whilst circles denote outliers. Different letters indicate significant differences as determined by the Wilcoxon rank sum test using the Benjamini—Hochberg method to adjust for multiple testing (*P*<0.05). The full *P*_adj_ values are shown in Supplementary Table S3.

To investigate whether CUL plays a role in virulence, we constructed *F. graminearum* mutant strains in the PH-1 background by deleting the longiborneol synthase gene *CLM1* (FGSG_10397). CUL production was completely abolished in the *clm1* deletion strains and they produced slightly higher amounts of DON than PH-1 on 2-SM medium (Supplementary Table S1). The subsequently generated marker-less *clm1::loxP* mutant strains GWCUL-37.37 and GWCUL-41.41 were used for further experiments. The wheat cultivar Apogee was infected with these strains, with the wild-type PH-1, and with a transformation negative control (PH-1t) in three independent infection experiments, all of which indicated that the CUL-deficient mutant strains are less aggressive on wheat than the parental PH-1 (Fig. 1A–C). Pairwise statistical analyses of the end-points (Supplementary Fig. S3) showed that not all comparisons with the wild type were significantly different at the α=0.05 level; however, the overall results of the three independent replications clearly suggested that the *clm1* mutants differed from the PH-1 controls (Supplementary Table S2). This was further investigated by calculating the rAUDPC, which showed that disease build-up was significantly different across the three experiments (Fig. 1D; Supplementary Table S3). At the end of two of the experiments (Fig. 1B, C), wheat ears were harvested and analysed for their contents of DON, D3G, CUL, and CULGs (the method used could not distinguish between CUL-8- and CUL-11-glucoside). The ears infected with the *clm1* deletion strains did not contain any CUL or CULGs, while both compounds were detected in ears infected with PH-1 (Supplementary Table S4). Meanwhile, the ears contained a higher DON content in the wild-type infections (significant for GWCUL-37.37), while D3G concentrations were comparable across all experiments. However, the D3G/DON ratios were significantly increased in *clm1*-infected ears compared with those infected with the wild-type strain PH-1, indicating an interference of CUL with the glucosylation of DON in wheat.

### Culmorin interferes with DON-glucosylation in wheat roots

**Fig. 2.**
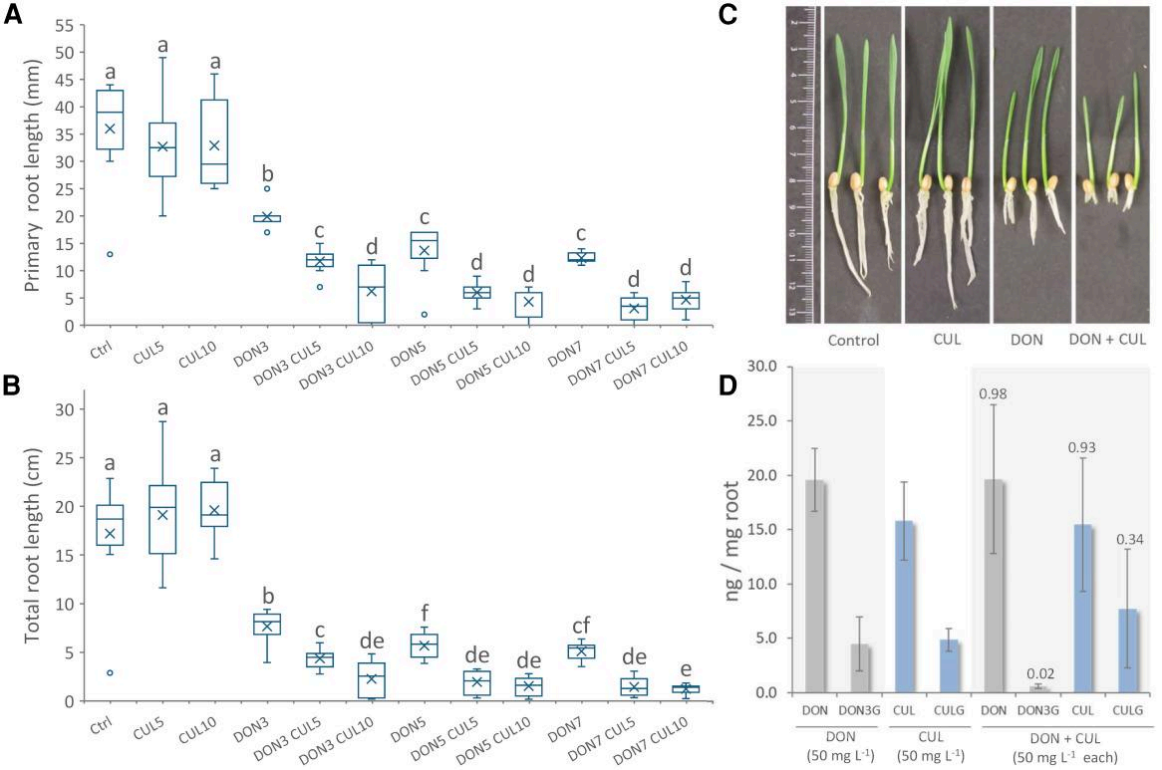
Impact of deoxynivalenol (DON) and culmorin (CUL) on roots of seedlings of the wheat cultivar Apogee. (A, B) Seedlings were grown for 96 h in media containing different concentrations of DON and/or CUL: the numbers on the *x*-axis refer to the concentrations in mg l^−1^; Ctrl, control (no CUL or DON). (A) Primary root length and (B) total root length of the seedlings after 96 h (*n*=10); × denotes the arithmetic mean whilst circles denote outliers. Different letters indicate significant differences as determined by the Wilcoxon rank sum test using the Benjamini—Hochberg method to adjust for multiple testing (*P*<0.05). The full *P*_adj_ values for (A) and (B) are shown in Supplementary Tables S5 and S6, respectively. (C) Representative images of seedlings after 96 h growth in medium containing 5 mg l^−1^ DON and/or CUL. (D) Concentrations of DON, DON-3-glucoside (D3G), CUL, CUL-glucoside (CULG) in roots extracted after 24 h incubation in medium with 50 mg l^−1^ DON and/or 50 mg l^−1^ CUL. Data are means (±SD), *n*≥4. The results of two-sample, two-tailed *t*-tests comparing the concentrations between the DON+CUL treatment and the corresponding single DON or CUL treatment are shown.

Root elongation assays have previously been established as toxicity assays for DON and other trichothecenes in Arabidopsis (Masuda *et al*., 2007), *B. distachyon* (Pasquet *et al*., 2016), and wheat (Wipfler *et al*., 2019). We compared the root length of Apogee wheat seedlings after 96 h of growth on agarose medium containing CUL and/or DON (Fig. 2A–C). DON caused a significant reduction of both primary (longest root) and total root length (Supplementary Tables S5, S6). While CUL alone did not cause a significant change, application together with DON significantly increased the degree of reduction of root length, and thus the toxicity of DON, confirming previous observations (Wipfler *et al*., 2019).

Analysis of root extracts prepared after incubation of seedlings with DON and/or CUL confirmed previous findings (Weber *et al*., 2018) that wheat is able to glucosylate CUL (Fig. 2D). The presence of CUL did not inhibit DON uptake by the roots and caused a significant reduction of ∼6-fold in the D3G concentration in the roots, suggesting that the synergy observed with DON is related to impaired ability to detoxify DON by UGTs.

### Culmorin inhibits conjugation of DON by UGTs

The above results indicated that CUL contributes to virulence and that its presence has a negative impact on DON-glucosylation by wheat UGTs, raising the question of whether CUL interferes with DON detoxification by acting as a potential competitive substrate or inhibitor of UGTs. In order to examine whether CUL interacts with UGTs, we examined the UGTs HvUGT13248 (barley), BdUGT5g03300 (*B. distachyon*), and OsUGT79 (rice), which have previously been characterized as efficiently detoxifying DON by glucosylation at position C3-OH (Michlmayr *et al*., 2018).

Simultaneous incubation of DON with CUL or CUL-conjugates (equimolar or at five-fold excess) clearly showed that they both reduced DON-glucosylation rates of all the investigated UGTs (Table 1). The D3G synthesis rates of HvUGT13248 and OsUGT79 were reduced by CUL and CUL-11-acetate while the glucosides and sulfates had moderate to no effect. BdUGT5g03300 was almost inactive (rate reduced to <1%) when CUL and CUL-11-acetate were present equimolar with DON. Even the glucosides and sulfates (except for CUL-8,11-disulfate) had a strong inhibitory effect on DON-glucosylation by BdUGT5g03300.

**Table 1.** Glucosylation rates of deoxynivalenol (DON) by UDP-glucosyltransferases from different species in the presence of culmorin (CUL) and culmorin-conjugate inhibitors, expressed as a percentage of the control

	BdUGT5g03300		HvUGT13248		OsUGT79	
	Concentration of inhibitor added (µM)					
Inhibitor	25	125	25	125	25	125
Control	100	100	100	100	100	100
Culmorin	0.84±0.03	0.19±0.01	59±6	13±<1	64±3	33±4
11-Acetyl-culmorin	0.83±0.01	0.12±<0.01	53±1	8.9±0.1	77±3	27±0
Culmorin-8-glucoside	5.7±<0.1	0.93±0.02	92±2	81±3	97±7	91±7
Culmorin-11-glucoside	2.5±<0.1	0.48±0.03	85±1	41±1	96±7	115±5
Culmorin-8-sulfate	12±<0.1	2.5±<0.1	97±4	79±1	105±1	89±4
Culmorin-11-sulfate	1.7±<0.1	0.30±0.02	90±2	49±3	91±7	61±2
Culmorin-8,11-disulfate	95±1	78±1	98±2	98±2	103±2	99±7

All assays were conducted with 25 µM DON and 10 mM UDP-glucose. The control consisted of the assay solution without an inhibitor. DON-3-glucoside synthesis was quantified by LC-MS/MS. Data are means (±SD) of *n*=3 assays.

### Culmorin glucosylation by UGTs

To clarify whether the reduction of activity with DON was a result of CUL competing with DON as a substrate, we compared the specific activities of the UGTs with DON and CUL at 25 µM each (Table 2). The results indicated that BdUGT5g03300 was almost inactive with CUL. Even after several hours of incubation, only traces of CUL-glucoside were detectable by LC-MS/MS. The specific activity with CUL was roughly estimated to be ∼1000-fold below the value obtained with DON. By contrast, HvUGT13248 glucosylated both compounds at comparable rates. Further kinetic analysis of HvUGT13248 with CUL revealed a high affinity with a *K*_M_ of 27±4 µM CUL and a *V*_max_ of 18.2±0.8 nmol min^−1^ mg^−1^ (Supplementary Fig. S4). Comparing the *V*_max_/*K*_M_ ratios, the catalytic efficiency of HvUGT13248 with CUL was comparable to or even higher than with DON (*K*_M_=1.8 mM DON and *V*_max_=0.18 µmol min^−1^ mg^−1^).

**Table 2.** Specific activities of UDP-glucosyltransferases from different species with deoxynivalenol (DON) and culmorin (CUL) both at 25 µM

	DON	CUL
UGT	Specific activity (nmol min^−1^ mg^−1^)	
BdUGT5g03300	1.07±0.03	≈ 1×10^−3^
HvUGT13248	8.77±0.52	6.94±0.52
OsUGT79	159±33	1.85±0.05
OsUGT79 Q202A*	161±46	2.23±0.08
OsUGT79 H122A/L123A/Q202A*	6.60±0.31	2.89±0.10

^*^Active site mutants of OsUGT79 (Wetterhorn *et al*., 2017). Assays were conducted with 10 mM UDP-glucose. The results refer to glucoside formation as quantified by LC-MS/MS. Data are means (±SD) of *n*=3 assays.

OsUGT79 glucosylated CUL at a low rate of 1–2% compared with DON (Table 2). We also investigated two active-site mutants that were previously created to expand the substrate range of OsUGT79 with trichothecenes. With DON, OsUGT79 Q202A is reported to behave in a kinetically similar way to the wild type enzyme, while the triple-mutant OsUGT79 H122A/L123A/Q202A has reduced affinity for DON, but also the broadest substrate range and accepted C4-acetylated trichothecenes (Wetterhorn *et al*., 2017). Here, we found that the wild type OsUGT79 and both mutants displayed similar conversion rates with CUL, while the catalytic rates observed with DON clearly differed (Table 2).

### Inhibition kinetics of UGTs with DON and CUL

To gain further insights into the mechanism of inhibition, we tested the influence of CUL on the reaction kinetics of all UGTs with DON and UDP-glucose. It should be noted that the assay we used quantifies released UDP via an enzymatic cascade causing NADH oxidation and does not distinguish between glucosylation of DON or CUL. To compensate for this, the blank reactions (‘0 mM DON’) also contained CUL. The estimates obtained in this way for the inhibition constants of competitive (*K*_ic_, Equation 3) and uncompetitive inhibition (*K*_iu_, Equation 4) are shown in Table 3.

**Table 3.** Influence of culmorin (CUL) on the kinetics of different UDP-glucosyltransferases with deoxynivalenol (DON) and UDP-glucose

UGT	Deoxynivalenol (1 mM UDP-glucose, 1–17 mM DON)				UDP-glucose (5 mM DON, 1–5 mM UDP-glucose)	
	*V*_max_ (µmol min^−1^ mg^−1^)	*K*_M_ (mM DON)	*K*_ic_ (µM CUL)	*K*_iu_ (µM CUL)	*K*_M_ (mM UDP-glc)	*K*_iu_ (µM CUL)
BdUGT5g03300	0.14	1.1	<1	–	0.19	1.4
HvUGT13248	0.18	1.8	56	–	0.33	91
OsUGT79	0.63	0.19	146	208	0.21	124
OsUGT79 Q202A*	0.85	0.29	16	56		
OsUGT79 H122A/L123A/Q202A*	0.26	1.3	65	217		

^*^Active site mutants of OsUGT79 (Wetterhorn *et al*., 2017). *K*_ic_, constant of competitive inhibition; *K*_iu_, constant of uncompetitive inhibition. Coupled enzyme assays were used to detect released UDP via NADH oxidation.

**Fig. 3.**
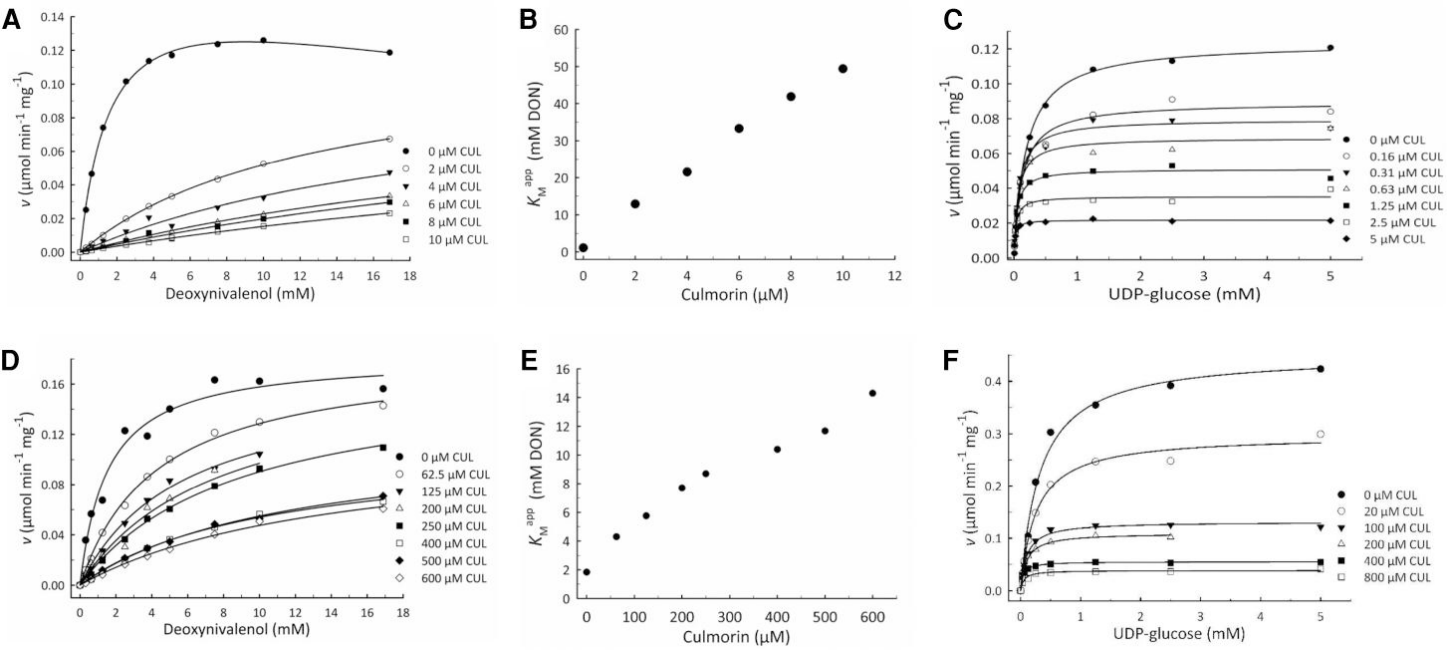
Influence of culmorin (CUL) on the kinetics of deoxynivalenol (DON) glucosylation by the UDP-glucosyltransferases BdUGT5g03300 (*Brachypodium distachyon*) and HvUGT13248 (*Hordeum vulgare*). (A) Effects of different concentrations of CUL on BdUGT5g03300 activity with DON in the presence of 1 mM UDP-glucose. (B) Apparent *K*_M_ (*K*_M_^app^) values for BdUGT5g03300 with DON plotted against CUL concentration. (C) Effects of different concentrations of CUL on BdUGT5g03300 activity with 5 mM DON at different UDP-glucose concentrations. (D) Effects of different concentrations of CUL on HvUGT13248 activity with DON in the presence of 1 mM UDP-glucose. (E) Apparent *K*_M_ values for HvUGT13248 with DON plotted against CUL concentration. (F) Effects of different concentrations of CUL on HvUGT13248 activity with 5 mM DON at different concentrations of UDP-glucose.

BdUGT5g03300 activity with DON was competitively inhibited with an increase of ∼50-fold of apparent *K*_M_ with DON at 10 µM CUL (Fig. 3A, B). *K*_ic_ was estimated to be in the low µM range (Table 3). Compared with the *K*_M_ of 1.1 mM DON, this implies remarkably strong binding of CUL. Inhibition kinetics with UDP-glucose as the variable substrate (5 mM DON) indicated an uncompetitive mechanism (Fig. 3C) with a decrease of both *V*_max_ and *K*_M_. *K*_iu_ with UDP-glucose was estimated at 1.4 µM CUL and it fell within the same concentration range as *K*_ic_ obtained with DON. The corresponding model of uncompetitive inhibition (Equation 4) assumes that the uncompetitive inhibitor binds only to the enzyme–substrate complex (i.e. UDP-glucose bound to BdUGT5g03300). Accordingly, our results imply that CUL binds with high affinity (*K*_D_≈1 µM level) in an unproductive position/orientation at the acceptor binding site of BdUGT5g03300 when UDP-glucose is already present at the donor-binding site.

Consistent with our observation that CUL was conjugated by HvUGT13248 with comparable efficiency to that of DON, inhibition of DON-glucosylation clearly followed a competitive mechanism with an increase of ∼10-fold of apparent *K*_M_ of DON at 600 µM CUL (Fig. 3D, E). The estimated *K*_ic_ (56 µM CUL) fell within a similar concentration range as *K*_M_ with CUL (25 µM). As observed with BdUGT5g03300, inhibition was uncompetitive with UDP-glucose (Table 3; Fig. 3F).

OsUGT79 had low activity with CUL and was only moderately inhibited by it. Nevertheless, it was interesting from a kinetic point of view because in assays without CUL, OsUGT79 displayed pronounced substrate inhibition by DON (Supplementary Fig. S5A). The Haldane model for uncompetitive substrate inhibition (Equation 2) is usually used to fit such data. It is assumed that the substrate can bind to the already formed enzyme–substrate complex at a second site and acts as inhibitor, for example by preventing the product from leaving (Bisswanger, 2015). The substrate inhibition by DON that we observed was reduced with increasing CUL concentrations, suggesting that CUL competes with DON for the hypothetical substrate inhibition site. CUL further caused a clear decrease in predicted *V*_max_. Although a slight increase of *K*_M_ was apparent (Supplementary Fig. S5B), this remained within a narrow concentration range and *K*_M_ remained effectively unchanged. This behavior resembled non-competitive inhibition, or mixed inhibition (Equation 5) where the inhibitor binds independently of whether the substrate is already bound at the active site (*K*_ic_≈*K*_iu_). The *K*_i_ values of CUL obtained with OsUGT79 (Table 3; Supplementary Fig. S5C, D) appear to confirm this model, although a completely non-competitive mechanism is unlikely, as CUL is also a substrate of OsUGT79. As in the cases of HvUGT13248 and BdUGT5g03300, inhibition with respect to UDP-glucose followed an uncompetitive mechanism (Supplementary Fig. S5G). Compared to the wild type OsUGT79, the estimated *K*_i_ values indicated even stronger binding of CUL by OsUGT79 Q202A and the competitive component was clearly increased (Table 3; Supplementary Fig. S5C, D). The triple-mutant OsUGT79 H122A/L123A/Q202A did not show substrate inhibition by DON (*K*_M_=1.3 mM DON) in the concentration range that we used (≤16 mM DON), and the competitive character of inhibition by CUL clearly dominated (Supplementary Fig. S5E, F).

### Inhibition of BdUGT5g03300 by CUL in *B. distachyon* is associated with the synergy of CUL with DON

Because of its strong inhibition by CUL and the fact that BdUGT5g03300 is crucial for *Fusarium*/DON resistance in *B. distachyon* (Pasquet *et al*., 2016), we next investigated the combined effect of DON and CUL on *B. distachyon* to clarify whether the effect observed on BdUGT5g03300 *in vitro* could be connected to DON-glucosylation *in vivo*. Compared to the wheat cultivar Apogee, *B. distachyon* Bd21-3 appeared to be more tolerant to DON. While Apogee already showed impaired root growth at 3 mg l^−1^ DON (Fig. 2), Bd21-3 was either not affected or barely affected between 2–8 mg l^−1^ DON, and root growth was observed up to 16 mg l^−1^ (Supplementary Fig. S6). As observed with wheat, the presence of CUL alone did not elicit any significant effect on root length; however, CUL caused a clear dose-dependent synergistic effect with 2 mg l^−1^ DON when applied at concentrations up to 2 mg l^−1^ (Supplementary Fig. S6; Supplementary Table S7).

**Fig. 4.**
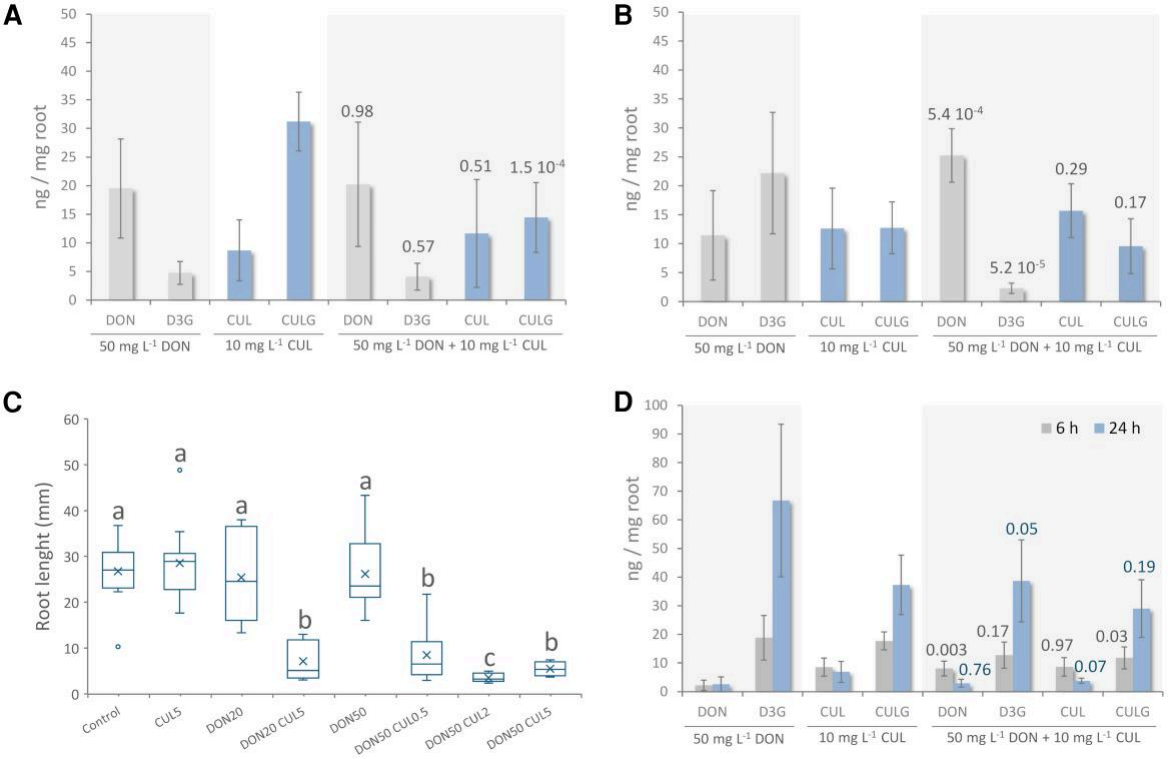
Impact of deoxynivalenol (DON) and culmorin (CUL) on roots of *Brachypodium distachyon* seedlings. (A, B) Seedlings were incubated for 24 h in liquid media with 50 mg l^−1^ DON and/or 10 mg l^−1^ CUL, after which the concentrations of DON, DON-3-glucoside (D3G), CUL, and CUL-glucoside (CULG) in the roots were determined. (A) Roots of TILLING mutant 6829-7 with a truncated version of the BdUGT5g03300 gene (W345*) and (B) roots of TILLING mutant 6829-3 carrying the wild-type BdUGT5g03300 allele. Data are means (±SD), *n*=10. The results of two-sample, two-tailed *t*-tests comparing the concentrations between the DON+CUL treatment and the corresponding single DON or CUL treatment are shown. (C) Root lengths of seedlings of the BdUGT5g03300 overexpression line OE-10R14 grown for 6 d in solid medium containing different concentrations of DON and/or CUL: the numbers on the *x*-axis refer to the concentrations in mg l^−1^; Ctrl, control (no CUL or DON). × denotes the arithmetic mean, whilst circles denote outliers; *n*≈10. Different letters indicate significant differences as determined by the Wilcoxon rank sum test using the Benjamini—Hochberg method to adjust for multiple testing (*P*<0.05). The full *P*_adj_ values are shown in Supplementary Table S8. (D) Seedlings of the OE-10R14 line were incubated for either 6 h or 24 h in liquid media with 50 mg l^−1^ DON and/or 10 mg l^−1^ CUL, after which the concentrations of DON, DON-3-glucoside (D3G), CUL, and CUL-glucoside (CULG) in the roots were determined. Data are means (±SD), *n*=5. The results of two-sample, two-tailed *t*-tests comparing the concentrations between the DON+CUL treatment and the corresponding single DON or CUL treatment at each time-point are shown.

To investigate the potential involvement of BdUGT5g03300, we analysed DON and CUL glucosylation in the roots of the *B. distachyon* TILLING mutant 6829-7, which carries a truncated (W345*) BdUGT5g03300 gene (Pasquet *et al*., 2016). The TILLING line 6829-3 that carries the wild-type BdUGT5g03300 allele was used as a control. Roots were incubated for 24 h in MS medium containing 50 mg l^−1^ DON and/or 10 mg l^−1^ CUL. The results showed that D3G synthesis in the mutant was low and it was not further reduced in the presence of CUL (Fig. 4A). In the wild type, D3G synthesis was clearly functional but suppressed in the presence of CUL, with concentrations in the DON+CUL treatment similar to those in the mutant (Fig. 4B). This indicated that BdUGT5g03300 is mainly responsible for D3G synthesis and is inhibited in the presence of CUL *in vivo*. We further investigated the BdUGT5g03300 overexpressing line OE-10R14 that has previously been shown to be highly DON/*Fusarium* resistant (Pasquet *et al*., 2016). In root elongation assays, this line tolerated up to 50 mg l^−1^ DON without significant reduction in length (Fig. 4C; Supplementary Table S8). Remarkably, addition of CUL even at low levels (0.5 mg l^−1^) to 50 mg l^−1^ DON caused a severe breakdown of resistance to DON. Analysis of root extracts taken after 6 h and 24 h incubation indicated high D3G formation capacity in the overexpression line (Fig. 4D). The presence of CUL significantly reduced D3G synthesis at 24 h, but not as drastically as would have been expected (∼58.6%). Our elongation and concentration assays were conducted at different time-points and under different CUL and DON treatments, and more detailed investigations are needed to clarify the time-dependent kinetics of DON/CUL glucosylation in the overexpression line. Nevertheless, our results clearly suggest that the tolerance gained by overexpression of BdUGT5g03300 is reduced in the presence of CUL.

We also conducted a preliminary infection experiment on the OE-10R14 line to investigate the effect of CUL deficiency in *F. graminearum* resulting from knock out of *CLM1*. After 7 d, disease severity was significantly lower in plants infected with the knockout strains compared with those infected with the wild type PH-1 (Supplementary Fig. S7; Supplementary Table S9). After 14 d, the infection had spread throughout the whole spike in all the treatments such that no significant differences were present; however, fungal DNA (as determined by qPCR of 18S rDNA) remained lower in the OE-10R14 spikelets infected with the knockout strains and they had a higher D3G/DON ratio (Supplementary Table S10), confirming the effect of CUL on OE-10R14.

### BdUGT5g03370 and BdUGT5g03380 glucosylate CUL

The above results further indicated that *Brachypodium* is able to glucosylate CUL and suggested that BdUGT5g03300 is not responsible. TILLING line 6829-7 (BdUGT5g03300 W345*) showed even higher CUL glucosylation than line 6829-3 carrying the intact allele (Fig. 4B). BdUGT5g03300 is member of a DON/*Fusarium*-responsive gene cluster containing six UGT genes designated BdUGT5g02780, BdUGT5g03300, BdUGT5g03370, BdUGT5g03380, BdUGTg03390 and BdUGT5g03400 (Schweiger *et al*., 2013a), of which only BdUGT5g03300 possesses activity with DON. BdUGT02780 is active with the trichothecene toxin nivalenol (NIV) but not with DON, and the functions of the other cluster members are unknown. Tentative assays with protein extracts of yeast expressing these UGTs indicated that BdUGT5g03370 and BdUGTg03380, both of which are highly expressed upon DON treatment/*Fusarium* infection (Schweiger *et al*., 2013a), are capable of glucosylating CUL (Supplementary Table S11). We further expressed both these UGTs in *E. coli* and estimated the kinetics of the one-step purified enzymes, which showed that BdUGT5g03370 glucosylated CUL with a *K*_M_ of 59±7 µM CUL and *V*_max_ of 0.57±nmol min^−1^ mg^−1^ whilst BdUGT5g03380 had a *K*_M_ of 150 µM CUL and *V*_max_ of 0.43±0.03 nmol min^−1^ mg^−1^ (Supplementary Fig. S8).

## Discussion

Fungi produce a multitude of secondary metabolites including polyketides, alkaloids, non-ribosomal peptides, and terpenes that might provide them with an ecological advantage (Keller *et al*., 2005; Brakhage, 2013). The potential functions of such metabolites are diverse and, in many cases, unknown. The increasing sensitivity of LC-MS/MS-based multi-methods has revealed that most food and feed commodities contain cocktails of different mycotoxins (Alassane-Kpembi *et al*., 2017). The toxicities of such mixtures are potentially complex, and studying the interactions between them is a challenging discipline in toxicology. Simultaneous exposure to different toxins can result in additive, synergistic (‘stronger-than-expected’) or antagonistic effects that can also depend on concentrations. For example, trichothecene toxins (DON, NIV, and acetylated derivatives) exert synergistic cytotoxicity at low doses, but additive to antagonistic toxicities at higher doses (Severino *et al*., 2006; Alassane-Kpembi *et al*., 2015).

Culmorin is a somewhat enigmatic *Fusarium* metabolite as it persistently co-occurs with DON at high levels but does not appear to possess an obvious function. The fact that the expression of biosynthetic genes of both metabolites is co-regulated under trichothecene-inducing conditions strongly suggests a functional link. Furthermore, CUL originates from the same biosynthetic pool as DON, and it is reasonable to hypothesize a complementary function. Previous studies have already indicated a possible synergy of CUL with DON in plants and hypothesized that CUL might interfere with DON-detoxification (Wang and Miller, 1988; Dowd *et al*., 1989; Wipfler *et al*., 2019). CUL also suppresses DON-glucuronidation by human glucuronosyltransferases in liver microsomes (Woelflingseder *et al*., 2019).

Here, we provide evidence that CUL-deficient *F. graminearum* strains are less aggressive on wheat (Fig. 1). We further show that the presence of CUL interferes with the detoxification of DON by conjugation to D3G in both wheat and *B. distachyon* (Figs 2, 4), indicating that CUL might contribute to *Fusarium* virulence by acting synergistically with DON.

Glucosylation is an important phase II conjugation reaction in plant secondary metabolism that alters solubility and chemical activity of bioactive compounds. It is involved in homeostasis of endogenous metabolites including defence metabolites and phytohormones, but also in the detoxification of xenobiotics (Lim and Bowles, 2004; Gachon *et al*., 2005; Bowles *et al*., 2006). Plant glucosylation of small molecules is catalysed by UGTs assigned to family 1 in the Carbohydrate-Active enZYme Database (http://www.cazy.org), which currently lists 138 glycosyltransferase families (Campbell *et al*., 1997; Coutinho *et al*., 2003). Family-1 UGTs are composed of two Rossman folds and the active site is located in a cleft between the two domains (Qasba *et al*., 2005; Lairson *et al*., 2008). UGTs are usually highly specific for the donor substrate (‘glycon’; i.e. UDP-glucose) that binds to the highly conserved plant secondary product glycosyltransferase (PSPG) motif located at the C-terminal domain, which is considered to be the signature motif of family-1 UGTs (pfam00201). The less conserved N-terminal domain interacts with the acceptor substrate (aglycon). Although some members are specific for the acceptor substrate, UGTs are generally promiscuous towards the aglycon (Gachon *et al*., 2005; Osmani *et al*., 2009), and a prerequisite for catalysis is the correct positioning of the functional group amenable for glycosylation (Osmani *et al*., 2009).

D3G, previously considered to be a ‘masked mycotoxin’, frequently co-occurs with its parental toxin to a variable extent (Berthiller *et al*., 2013), indicating that glucosylation of DON is a relevant plant defence mechanism. Several plant UGTs with the ability to convert DON into the non-toxic D3G have been identified (Michlmayr *et al*., 2018; Gharabli *et al*., 2023). Following our observation that CUL inhibits DON-glucosylation in wheat, a future research question will be to investigate wheat UGTs with regards to their inhibition by CUL. However, although several candidate UGTs of hexaploid wheat (*Triticum aestivum*) have been proposed to be involved in *Fusarium* resistance, there is little evidence for their actual contribution to DON detoxification. The DON-responsive TaUGT3 is reported to increase tolerance to DON (Lulin *et al*., 2010), but DON-glucosylation could not be confirmed with a DON-sensitive yeast strain (Schweiger *et al*., 2010). Another pathogen-inducible UGT, TaUGT12887, is only weakly active with DON (Schweiger *et al*., 2013b). Traes_2BS_14CA35D5D, a homolog of BdUGT5g03300, provides *Fusarium* resistance *in planta*, but has not yet been biochemically characterized with DON (Gatti *et al*., 2018). TaUGT6 is another candidate that has been proposed to be involved in resistance, but the purified protein is described as only ‘active to some extent’ with DON (He *et al*., 2020).

In the absence of biochemically validated DON-detoxifying UGTs from wheat, in this study we investigated the previously characterized BdUGT5g03300 (*B. distachyon*), OsUGT79 (rice), and HvUGT13248 (barley) as these possess sufficient activities with DON to conduct kinetic assays (Michlmayr *et al*., 2018). Barley HvUGT13248 is DON-inducible (Gardiner *et al*., 2010) and confers resistance when expressed in Arabidopsis and wheat (Li *et al*., 2015; Li *et al*., 2017; Mandalà *et al*., 2019). Loss of function of HvUGT13248 leads to reduced resistance to the spread of *Fusarium* in barley, demonstrating the important role of detoxification in host defence (Bethke *et al*., 2023). In addition, BdUGT5g03300 (‘Bradi5g03300’) is important for *Fusarium* resistance of the monocot model plant *B. distachyon* (Pasquet *et al*., 2016) and provides resistance to wheat upon heterologous overexpression (Gatti *et al*., 2019). Disruption of BdUGT5g03300 in *B. distachyon* by TILLING leads to increased susceptibility to DON and reduced resistance to *F. graminearum* (Pasquet *et al*., 2016). OsUGT79 in rice was identified by homology to both BdUGT5g03300 and HvUGT13248. It is highly active with DON and its 3D structure yields crucial insights into trichothecene accommodation at the active site (Wetterhorn *et al*., 2016, 2017). We therefore used these UGTs as case-study models to investigate whether CUL is capable of impeding DON-detoxification by serving as an alternative substrate or inhibitor. Our results indicated that CUL can in fact interfere with DON-detoxification by UGTs, although the effect was clearly variable in magnitude and mechanism. Inhibition by CUL was uncompetitive with UDP-glucose in all cases (Table 3), which indicated that the inhibitor can only bind when the enzymes are already present in a complex with UDP-glucose. This is consistent with the fact that UGTs undergo conformational changes upon substrate binding and the sugar donor usually binds first (Breton *et al*., 2012). It is possible that UDP-glucose induces conformational changes to make the corresponding sites accessible.

Our results further suggest that CUL inhibits UGTs by binding to sites that recognize DON although it does not necessarily mimic DON as a substrate analogue. This resulted in remarkably strong unproductive binding/inhibition of BdUGT5g03300 and competitive inhibition of HvUGT13248 by CUL serving as an alternative substrate. In the case of OsUGT79, CUL appeared to compete with the alternative binding site that DON occupies to exert substrate inhibition (Supplementary Fig. S5). According to the log*P* values published on PubChem (https://pubchem.ncbi.nlm.nih.gov/, accessed September 2025), CUL is strongly hydrophobic (XLogP3-AA = 3.3) while DON (XLogP3-AA = –0.7) is hydrophilic. Considering the functional ‘promiscuity’ of defence-related UGTs and the largely hydrophobic nature of UGT acceptor binding pockets (Osmani *et al*., 2009), it might not be surprising that a hydrophobic metabolite of relatively small mass (CUL 238 g mol^–1^) can bind—in some cases even with high affinity—to the active site of a UGT in a productive or unproductive orientation. From such a perspective, it can be hypothesized that other phase-II detoxification enzymes such as glutathione transferases with low selectivity as a ‘functional principle’ could be affected analogously. We have previously shown that tau class glutathione S-transferases (GSTs) from wheat are capable of conjugating DON as well (Michlmayr *et al*., 2025). Besides DON detoxification, both UGTs and GSTs play diverse catalytic roles in plant stress responses and these might likewise be affected by CUL. As demonstrated here, both wheat and *B. distachyon* were able to glucosylate CUL (Figs 2, 4), which would for example diminish its inhibitory activity on HvUGT13248. In contrast, the CUL-glucosides were also inhibitory to BdUGT5g03300, which implies that glucosylation catalysed by other UGTs such as BdUGT5g03370 and BdUGT5g03380 might not counteract its synergistic effect with DON. With respect to transgenic approaches to increase *Fusarium* resistance by overexpression of UGTs , this implies that OsUGT79 and HvUGT13248 might have advantages over BdUGT5g03300.

In conclusion, this study presents biochemical evidence that CUL might contribute to *Fusarium* virulence by inhibiting DON-detoxification by plant UGTs, resulting in a synergistic effect with DON. CUL production could allow the fungus to overcome transgenic or breeding efforts to increase *Fusarium* resistance by increasing DON-detoxification by glucosylation. The interplay between trichothecenes as *Fusarium* virulence factors and plant detoxification enzymes has presumably been ongoing for about 27 million years (O’Donnell *et al*., 2013). Our results imply that UGTs of different plant species not only had to co-evolve with structurally diverse virulence factors but also with co-produced inhibitors such as culmorin, and potentially many other compounds.

## Data Availability

The supplementary tables and figures, as well as the raw data used for the plots of all figures, are available at Zenodo at http://dx.doi.org/10.5281/zenodo.18849251 (Michlmayr *et al*., 2026).

## Supplementary data

The following supplementary data are available on Zenodo (http://dx.doi.org/10.5281/zenodo.18849251).

Table S1. Production of DON and CUL by *F. graminearum* PH-1 and *CLM1* knockout transformants on 2-SM medium.

Table S2. Statistical analysis of wheat infection scores (related to Fig. 1).

Table S3. Wilcoxon rank sum test of rAUDPC (related to Fig. 1).

Table S4. CUL, CULGs, DON, and D3G concentrations in infected wheat heads.

Table S5. Wilcoxon rank sum test of primary wheat root length after DON and CUL treatments (related to Fig. 2A).

Table S6. Wilcoxon rank sum test of total wheat root length after DON and CUL treatments (related to Fig. 2B).

Table S7. Wilcoxon rank sum test of *B. distachyon* total root length after DON and CUL treatments (related to Fig. S7).

Table S8. Wilcoxon rank sum test of total root length of BdUGT5g03300 overexpression *B. distachyon* line OE-10R14 after DON and CUL treatments (related to Fig. 4C).

Table S9. Statistical analysis of infection scores of BdUGT5g03300 overexpressing *B. distachyon* line OE-10R14 (related to Fig. S7).

Table S10. Fungal DNA, DON, D3G, CUL, and CULGs in infected *B. distachyon* OE-10R14.

Table S11. Screening of *B. distachyon* UDP-glucosyltransferases for activity with CUL.

Fig. S1. Deletion of *CLM1* in *Fusarium graminearum* PH-1.

Fig. S2. Experimental set-up of root elongation assays with wheat Apogee seedlings.

Fig. S3. Disease progression and box-plots of Apogee infection scores.

Fig. S4. Kinetics of HvUGT13248 with CUL.

Fig. S5. Inhibition kinetics of OsUGT79 with DON and CUL.

Fig. S6. Effects of exposure to DON and CUL on *B. distachyon* seedlings.

Fig. S7. Percentage infected spikelets of *B. distachyon* OE-10R14 line infected with *F. graminearum* PH-1 and *CLM1* knockout transformants.

Fig. S8. Kinetics of BdUGT5g03370 and BdUGT5g03380 with CUL.

## Abbreviations

CUL: culmorin
CULGs: CUL-8- and CUL-11-glucosides
DON: deoxynivalenol
D3G: deoxynivalenol-3-glucoside
TILLING: targeting-induced local lesion in genomes
UGT: UDP-glucosyltransferase
